# Algae Biomass Hydrogels for Enhanced Removal of Heavy Metal Ions

**DOI:** 10.3390/gels11030150

**Published:** 2025-02-20

**Authors:** Mingjie Zhao, Dadong Wang, Zhen Fan, Jian Lu, Yibo Li, Yongwei Zhang, Mingchen Lv, Min Sun, Wenji Wang

**Affiliations:** 1 Shanghai Landscape Architecture Design and Research Institute, 45 Xin Le Road, Shanghai 200031, China; m223zhao@uwaterloo.ca (M.Z.); lujian@wx.shlandscape.com (J.L.); liyibo_1563@163.com (Y.L.); zyw2004412@163.com (Y.Z.); 2 Shanghai Engineering Technology Research Center for Ecological Landscape Water Environment, 202 Wukang Road, Shanghai 200031, China; ctglwangdd@chengtou.com; 3 Department of Polymeric Materials, School of Materials Science and Engineering, Tongji University, 4800 Caoan Road, Shanghai 201804, China; fanzhen2018@tongji.edu.cn (Z.F.); 1910582@tongji.edu.cn (M.L.); 4 Graduate School of Architecture Planning and Preservation, Columbia University in the City of New York, 116th St & Broadway, New York, NY 10027, USA

**Keywords:** hydrogel, algae, biomass, metal removal, bioremediation

## Abstract

Heavy metal ion pollution in aquatic environments is a critical global issue, damaging ecosystems and threatening human health via bioaccumulation in the food chain. Despite promising progress with biosorbents, the development of environmentally friendly and stable heavy metal adsorbents requires further exploration. In this study, we present an algae-loaded alginate hydrogel as a composite adsorbent for heavy metals. The incorporation of algae enhanced the hydrogel’s adsorption capacity by 38.0%, 20.6%, and 27.1% for Cu^2+^, Cr^3+^, and Co^2+^, respectively. Additionally, the composite hydrogel demonstrated excellent stability and recyclability after adsorption, reducing the ecological risks associated with algae biomass usage. This algae-loaded alginate hydrogel offers an efficient and eco-friendly strategy for removing heavy metal ions from aquatic systems, highlighting its potential for environmental remediation applications.

## 1. Introduction

Heavy metal ion pollution in aquatic systems is a pressing environmental issue that has received much attention in recent decades [1]. Heavy metal ion pollution has deep ecological impacts, leading to changes in microbial community structure, including a reduction in biodiversity and the disruption of ecosystems [2]. The presence of heavy metal ions in water causes a serious threat to aquatic organisms [3]. Many of these metal ions could bioaccumulate in aquatic organisms, resulting in toxic effects that disrupt both physiological and reproductive processes [4]. In addition, heavy metal ions in water can disrupt the balance of aquatic ecosystems by inhibiting the growth of certain phytoplankton species while promoting the reproduction of others. This imbalance reduces dissolved oxygen levels, leading to the death of fish and other aquatic organisms [5]. In addition, through the process of biomagnification effect, heavy metal ion levels could rise along the food chain, causing risks to large aquatic predators and ultimately to humans [6]. The persistence and non-biodegradability of heavy metals in the environment highlight the urgent need for effective control of heavy metal ion pollution in water [7].

In recent years, numerous materials have been developed for the removal of heavy metal ions from aqueous systems, each with unique adsorption properties, such as porous materials, polymer hydrogels, and biosorbents [8]. The removal of heavy metal ions by these materials is achieved by adsorption or chelation effect, owing to their high specific surface area or inherent functional groups. Metal–organic frameworks (MOFs) are a typical porous material for metal ions adsorption, renowned for their tunable porous structures and high metal ion selectivity [9]. In addition, metal-based nanomaterials offer enhanced adsorptive capacities owing to their high surface reactivity at the nanoscale [10]. While these materials have significant advantages such as high adsorption capacity, high selectivity, and high regeneration potential, their disadvantages are also notable. The stability of some porous materials (e.g., MOFs) in aqueous solution is still a concern [11]. In addition, the possible secondary pollution caused by metal-based materials, the scalability of large-scale applications, and economic feasibility are all challenges that need to be addressed.

In addition to the above materials, polymeric materials and biosorbents for metal ion adsorption have received much attention in recent years [12]. These materials provide abundant functional groups that can effectively chelate metal ions to achieve metal ions adsorption [13]. Polymer hydrogels, characterized by their three-dimensional polymer networks, have emerged as a promising material for the remediation of heavy metal pollution in aqueous systems [14]. The inherent hydrophilicity of hydrogels provides them with significant water absorption and stability, offering a large surface area for interaction with metal ions. Functional groups in the polymer backbone of hydrogels, such as carboxyl, amine, and hydroxyl groups, can chelate or adsorb heavy metal ions, thereby facilitating their removal from contaminated water. At the same time, the flexibility of hydrogel synthesis allows for the incorporation of specific functional molecules or nanoparticles to improve selectivity and adsorption of target metal ions. In addition, the swellability of hydrogels allows for easy recycling and regeneration, making them an attractive and sustainable option for water purification [15]. Wang and his co-workers reported a poly(polyethylenimine/hydroxyethyl acrylate) hydrogel for Cu^2+^ removal and reached the adsorption capacity for Cu^2+^ at 40.00 mg/g [16]. Luo and his co-workers reported a crosslinked carboxymethyl chitosan hydrogel for Co^2+^ and reached the adsorption capacity at 72.84 mg/g [17]. More recently, Ouass and his co-workers reported a sodium polyacrylate hydrogel for Cr^3+^ removal, and achieved a maximum adsorption capacity of 269 mg/g in acidic aqueous solutions [18]. Although traditional polymer hydrogels have shown great potential for heavy metal ion removal, the combination with biomass can further enhance the adsorption applications of polymer hydrogels.

Alginate is a natural polysaccharide extracted from brown algae and a typical polymer for heavy metal ions removal [19]. Alginate possesses excellent biocompatibility and can be easily crosslinked by divalent cations to form alginate hydrogels. Alginate hydrogels are filled with carboxylic acid groups that help to adsorb and immobilize metal ions in water, offering excellent adsorption capability. In addition, given the renewability and biodegradation potential of alginate, alginate hydrogels represent a sustainable and environmentally friendly approach to the challenge of heavy metal contamination in water systems.

Algae biomass is a new class of biosorbents, a group of photosynthetic microorganisms capable of rapid proliferation in aquatic environments [20]. In recent years, algae biomass has attracted much attention in the field of environmental biotechnology, especially in the remediation of heavy metal polluted waters [21]. The cell surfaces of algae are rich in functional groups such as carboxyl, hydroxyl, and amino groups, which have the tendency to chelate or complex with heavy metal ions, thereby facilitating their adsorption and subsequent removal from aqueous solutions [22]. The adsorption of heavy metal ions using algal biomass provides a sustainable, cost-effective, and environmentally friendly alternative to traditional remediation techniques [23]. In addition, the regeneration and reuse potential of algal biomass further highlights its viability as a green adsorbent in water remediation applications [24]. In addition, the properties of easy accessibility and the rapid growth rate of algae make them a ready resource for large-scale biosorption processes. Further, biotechnological approaches have facilitated the modification of algal biomass to enhance its metal ion affinity and selectivity, thus leading to new approaches for the targeted removal of specific heavy metals ions [25].

However, there are still some risks associated with the employment of algae adsorbents: the employment of live algae in aquatic systems may result in large and uncontrolled blooms that may affect or even disrupt existing ecosystems [26]; the small size of algae makes them difficult to collect after adsorption of heavy metal ions, which remain in the aquatic ecosystem; and the introduction of algae may make them food for aquatic predators, thereby bioaccumulating in aquatic predators and even subsequently in the food chain. In this study, we developed an algae-loaded alginate hydrogel system that combined the strengths of biosorbents and polymer hydrogels for enhanced heavy metal ion removal from solutions. This hydrogel system presented several key advantages: (1) the alginate hydrogel exhibited a good heavy metal ion adsorption capacity, which was further improved after loading the algae; (2) the composited hydrogel has excellent water stability with macroscopic size, which facilitates the employment and recycling of the adsorbent; and (3) compared to simple algae biosorbents, the composited hydrogels avoid the risk of algae bloom and consumption by aquatic organisms, which minimizes their impact on the ecosystem. Our experimental results show that the composite hydrogel has an improved adsorption efficiency for metal ions, demonstrating its potential as a novel eco-friendly biosorbent.

## 2. Results and Discussion

**Figure 1:**
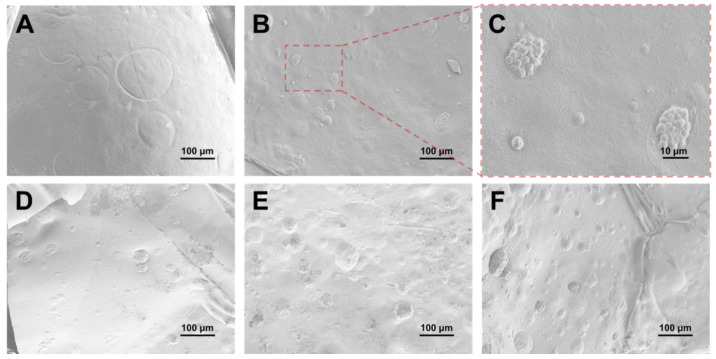
SEM images of (**A**) calcium alginate hydrogels without algae (0 mg algae hydrogel), (**B**) calcium alginate hydrogels with algae (1 mg algae hydrogel), and (**C**) enlarged SEM images of 1 mg algae hydrogel. (**D**–**F**) are SEM images of 1 mg, 2 mg, and 5 mg algae hydrogels, respectively.

Firstly, the algae-loaded calcium alginate hydrogel was prepared at room temperature [27]. The calcium alginate hydrogel without algae loading was denoted as 0 mg algae hydrogel, and the calcium alginate hydrogel with different algae loading ratios were denoted as 1 mg, 2 mg, and 5 mg algae hydrogels. Calcium ions facilitate the immobilization of algae within the hydrogel matrix by crosslinking alginate chains, ensuring stable integration and enhancing the accessibility of the algae’s functional groups for metal ion adsorption. Then, scanning electron microscopy (SEM) was used to confirm the successful loading of algae. Figure 1A showed the SEM image of 0 mg algae hydrogel, which exhibited a smooth and homogeneous surface. This smooth morphology was characteristic of the typical gelation process of sodium alginate in the presence of calcium ions. In contrast, Figure 1B shows the SEM image of a hydrogel loaded with algae (1 mg algae hydrogel). The presence of Chlorella vulgaris algae could be clearly identified, as small, spherical particles were visible within the hydrogel matrix. These particles corresponded to the algae cells that were successfully incorporated into the calcium alginate network. To further highlight the presence of algae in the hydrogel, Figure 1C presented an enlarged view of Figure 1B, offering a closer look at the distribution of algae within the hydrogel. Figure 1D–F shows the SEM images of the algae-loaded hydrogels with varying amounts of algae loading (1 mg, 2 mg, and 5 mg algae hydrogels). As the algae content increased, a broader distribution of algae became evident within the hydrogel. In Figure 1D, the 1 mg algae hydrogel showed a moderate presence of algae evenly distributed throughout the matrix. However, with the increase in algae loading to 2 mg and 5 mg, as shown in Figure 1D–F, the algae were more densely packed within the hydrogel, leading to a more pronounced presence within the hydrogel. The increased algae content resulted in a higher density of algae particles, which were more readily visible in the SEM images. These results confirmed the successful preparation of the algae-loaded hydrogels.

**Figure 2:**
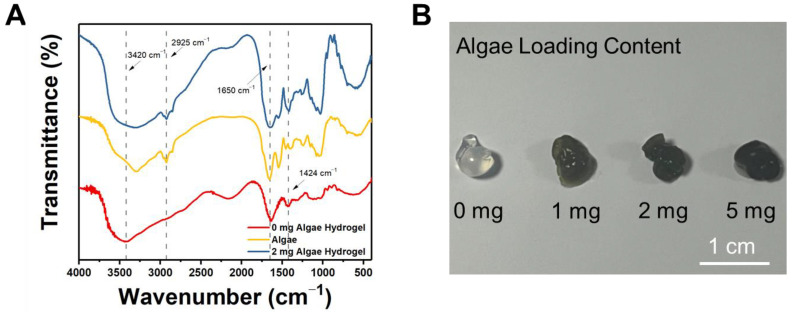
(**A**) FTIR results of calcium alginate hydrogel without algae (0 mg algae hydrogel), algae and calcium alginate hydrogel with 2 mg algae loading (2 mg algae hydrogel). (**B**) Images of calcium alginate hydrogel without algae and calcium alginate hydrogels containing 1 mg, 2 mg, and 5 mg algae.

To further confirm the successful incorporation of algae into the hydrogels, Fourier Transform Infrared Spectroscopy (FTIR) was performed, and the results were shown in Figure 2A. When comparing the spectra of algae, pure calcium alginate hydrogel (0 mg algae hydrogel) and the algae-loaded hydrogel (2 mg algae hydrogel), it can be seen that, although the algae-loaded hydrogel shares many characteristic peaks with the pure calcium alginate hydrogel, a distinctive characteristic peak appears at 2925 cm^−1^ that could be observed for both the pure algae and 2 mg algae hydrogel, which corresponds to the s alkyl stretching of the algae Chlorella vulgaris, confirming that the algae was successfully incorporated into the hydrogel matrix [28,29]. In addition, the peak at 3420 cm^−1^ was assigned to the hydroxyl stretching, and peaks at 1424 and 1650 cm^−1^ were assigned to the symmetric carbonyl stretching and carboxyl asymmetric stretching [30]. The FTIR spectrum of the pure calcium alginate hydrogel (0 mg algae hydrogel) exhibits characteristic peaks corresponding to the hydroxyl and carboxyl groups in the alginate polymer, which contribute to the adsorption of metal ions.

Figure 2B presented photographs of the prepared 0 mg, 1 mg, 2 mg, and 5 mg algae hydrogels, respectively. The hydrogels without algae loading appeared light-colored, which was typical of calcium alginate hydrogels. In contrast, the hydrogels with algae loading exhibited a dark green color, due to the green color of Chlorella vulgaris, with the color intensity increasing as the algae content raised. The darker color in the hydrogel correlated with the increased algae loading, providing a visible indication that the amounts of algae incorporated into the hydrogel increases with higher loading levels.

Therefore, both the FTIR spectra and photographic evidence demonstrated that Chlorella Vulgaris algae were successfully loaded into the calcium alginate hydrogels.

**Figure 3:**
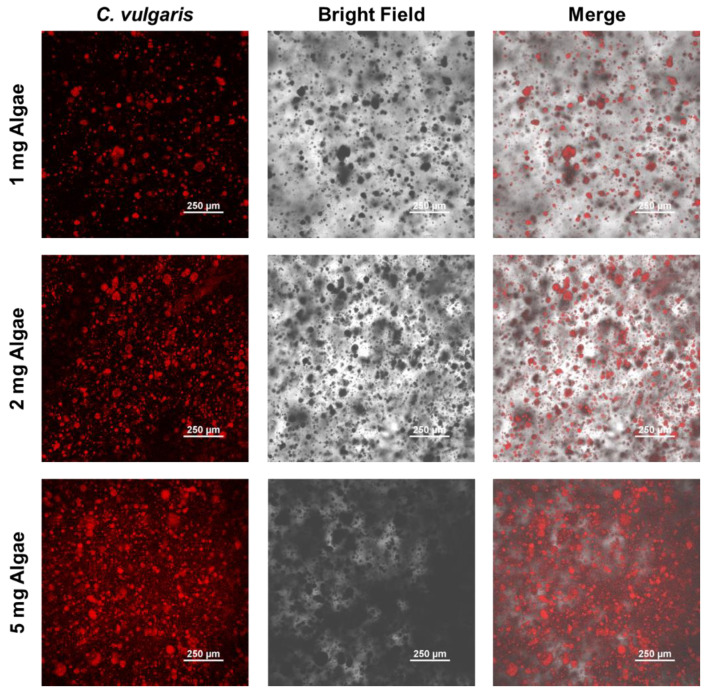
CLSM images of 1 mg, 2 mg, and 5 mg algae hydrogels.

To examine the distribution of algae within the hydrogels, Confocal Laser Scanning Microscopy (CLSM) was utilized, leveraging the intrinsic fluorescence of Chlorella vulgaris. The algae emitted a natural red fluorescence, primarily attributed to chlorophyll, which absorbs light and emits fluorescence under specific excitation wavelengths. Figure 3 presents the fluorescence images of Chlorella vulgaris within the calcium alginate hydrogels.

The red fluorescence signals clearly indicated the presence of Chlorella vulgaris throughout the hydrogels, demonstrating the effective and uniform integration of algae into the calcium alginate matrix. As the algae loading content increased, the fluorescence intensity became noticeably stronger, corresponding to the higher number of algae cells incorporated within the hydrogel. Additionally, the transparency of the hydrogels decreased with increasing algae content. This reduction in transparency aligned with the enhanced fluorescence, likely resulting from light scattering caused by the physical presence of more algae. As algae loading increased, the hydrogels appeared opaquer, further confirming the successful incorporation of larger quantities of algae.

These results provide strong evidence for the uniform distribution of Chlorella vulgaris within the calcium alginate hydrogels and confirm the effective incorporation of algae at varying loading levels.

After confirming the successful preparation of algae-loaded hydrogels, their adsorption capability for heavy metal ion removal was evaluated using Cu^2+^, Cr^3+^, and Co^2+^ as model metal ions. These ions are commonly found in industrial wastewater and pose significant environmental and health risks, making their removal essential for environmental remediation [16,17,18]. We chose Cr(III) as one of the metal models to demonstrate the capability of our hydrogels to absorb trivalent metal ions. Meanwhile, Cr(III) is also one of the heavy metal ions in industrial wastewater, which could be transformed into more toxic Cr(VI) under certain conditions [31,32]. Therefore, the removal of excess Cr(III) from wastewater is also important to reduce pollution. The interactions between alginate and metal ions are primarily driven by the chelation of the alginate’s carboxyl groups, while algae introduce additional functional groups (e.g., amino, hydroxyl, sulfhydryl) that enhance binding through electrostatic interactions, hydrogen bonding, and complexation.

**Figure 4:**
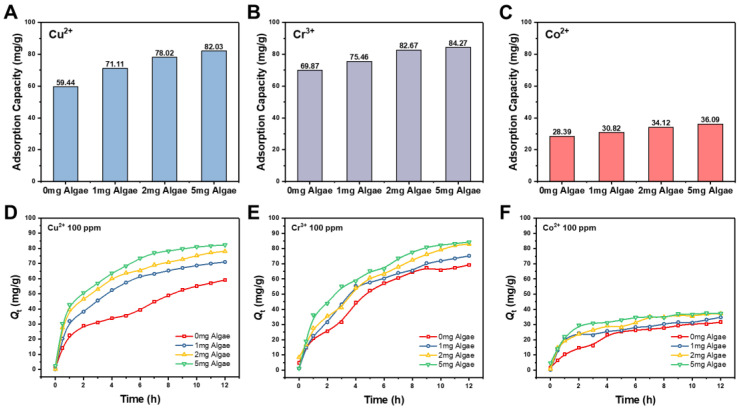
(**A**) Cu^2+^, (**B**) Cr^3+^, and (**C**) Co^2+^ adsorption capacities of 0 mg, 1 mg, 2 mg, and 5 mg algae hydrogels, respectively. Adsorption kinetics of Cu^2+^ (**D**), Cr^3+^ (**E**), and Co^2+^ (**F**) removal by 0 mg, 1 mg, 2 mg, and 5 mg algae hydrogels, respectively. (*Q*_t_: amount of adsorption capacity at time t).

Figure 4A–C presented the adsorption capacities of the hydrogels for Cu^2+^, Cr^3+^, and Co^2+^, respectively. Calcium alginate hydrogels (0 mg algae hydrogel) effectively adsorbed all three metal ions, primarily due to the chelating properties of alginate. The carboxyl and hydroxyl groups in alginate could coordinate with metal ions, facilitating metal removal from solutions. However, adsorption capacity varied by metal ion type, indicating differences in the interaction between alginate and each ion.

The incorporation of algae significantly enhanced the adsorption performance of the hydrogels, as shown in Figure 4A–C. Adsorption capacities increased with higher algae loading, from 59.44 mg/g (no algae loading) to 82.03 mg/g (5 mg algae loading) for Cu^2+^, from 69.87 mg/g (no algae loading) to 84.27 mg/g (5 mg algae loading) for Cr^3+^, and from 28.39 mg/g (no algae loading) to 36.09 mg/g (5 mg algae loading) for Co^2+^. These represent relative increases of 38.0% for Cu^2+^, 20.6% for Cr^3+^, and 27.1% for Co^2+^. The differences in adsorption enhancement were attributed to variations in the sensitivity of algae functional groups to different metal ions.

These results demonstrate that algae-loaded hydrogels significantly improved adsorption capacities compared to hydrogels without algae. This improvement is likely due to the combined effects of the alginate’s metal-binding ability and the additional adsorption sites provided by the algae. Algae cells contain diverse functional groups, such as amino acids, proteins, and polysaccharides, which interact with metal ions and further enhance the overall adsorption capacity of the hydrogels.

To further investigate the adsorption kinetics of the hydrogels for the three metal ions (Cu^2+^, Cr^3+^, and Co^2+^), experiments were conducted with an initial metal ion concentration of 100 ppm. Then, the adsorption kinetics of the hydrogels against the three metal ions were measured and are shown in Figure 4D–E.

Figure 4D shows that for Cu^2+^ adsorption, no algae-loaded hydrogels exhibited a rapid increase in adsorption capacity within the first 2 h. In contrast, algae-loaded hydrogels showed a longer rapid adsorption phase, lasting 5 to 6 h, which can be attributed to the additional adsorption sites provided by the algae. After this phase, adsorption slowed, reaching equilibrium at approximately 10 h.

For Cr^3+^ adsorption (Figure 4E), rapid adsorption was observed within the first 5 h for all hydrogels, with the algae-loaded hydrogels demonstrating greater adsorption increases compared to the pure calcium alginate hydrogels. Equilibrium was reached after about 9 h.

As shown in Figure 4F, Co^2+^ adsorption exhibited different kinetics. Algae-loaded hydrogels achieved rapid adsorption within 2 h, while pure calcium alginate hydrogels showed slower adsorption, requiring 4 h to reach rapid adsorption. Equilibrium was achieved for all hydrogels within 4–5 h.

Comparing the adsorption kinetics for the three metal ions, the algae-loaded hydrogels consistently exhibited faster adsorption rates and longer rapid adsorption phases than pure calcium alginate hydrogels. This enhancement is likely due to the additional functional groups provided by the algae, which increased the number of adsorption sites and influenced the dynamics of metal ion uptake. Based on the result from adsorption kinetics, the adsorption kinetics of metal ions belonged to pseudo-second-order kinetics, which indicated that metal ion removal by algae hydrogels was a chemisorption process [33].

The improved adsorption rate and extended rapid adsorption phase in algae-loaded hydrogels led to a higher overall adsorption capacity. The presence of algae enhanced the initial adsorption efficiency and sustained adsorption kinetics, allowing the hydrogels to capture more metal ions in a shorter time frame. These results underscore the synergistic role of algae in boosting the adsorption performance of the composite hydrogels.

**Figure 5:**
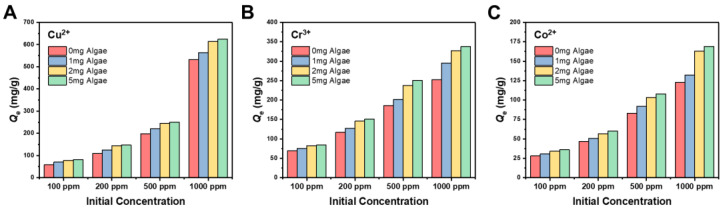
Equilibrium adsorption capacities (*Q*_e_) of 0 mg, 1 mg, 2 mg, and 5 mg algae hydrogels, respectively, at different initial concentrations (100 ppm, 200 ppm, 500 ppm, and 1000 ppm) of Cu^2+^ (**A**), Cr^3+^ (**B**), and Co^2+^ (**C**).

To investigate the equilibrium adsorption capacities of the hydrogels under varying conditions, experiments were performed with initial metal ion concentrations of 100 ppm, 200 ppm, 500 ppm, and 1000 ppm. Figure 5 illustrates the relationship between initial metal ion concentrations, algae loading, and the adsorption efficiency of the hydrogels.

As shown in Figure 5, the equilibrium adsorption capacities increased with higher initial concentrations for all metal ions and hydrogels. The increase in adsorption capacity of hydrogels with higher initial metal ion concentrations can be attributed to concentration gradient and adsorption equilibrium [33]. A higher initial concentration creates a larger concentration gradient between the solution and the hydrogel, which enhances the driving force for ion migration toward the adsorption sites. In addition, the adsorption equilibrium shifts with increasing concentrations. At higher initial concentrations, the equilibrium favors more ions binding to the hydrogel, resulting in higher adsorption capacities at equilibrium.

The incorporation of algae further enhanced adsorption capacities, with the effect directly proportional to the algae loading content. This indicates that the encapsulated algae actively participated in metal ion uptake.

Notably, the enhancement was most significant at higher initial metal ion concentrations, suggesting that algae not only provided additional adsorption sites but also improved the hydrogel’s capacity to bind larger quantities of metal ions under these conditions.

In summary, the increase in adsorption was closely linked to the algae loading content, confirming that algae played a pivotal role in enhancing the adsorption performance of the hydrogels. The pronounced effect at higher initial concentrations highlights the combined contributions of alginate and algae in improving the adsorption efficiency and capacity of the composite hydrogels.

## 3. Conclusions

In this study, we developed an algae-loaded alginate hydrogel system as an effective adsorbent for the removal of heavy metal ions. Various concentrations of algae were successfully incorporated into calcium alginate hydrogels, resulting in composite adsorbents with enhanced metal ion removal capabilities. The adsorption efficiency for Cu^2+^, Cr^3+^, and Co^2+^ increased by 38.0%, 20.6%, and 27.1%, respectively, compared to the control hydrogels. Additionally, the presence of algae was found to significantly improve both the adsorption rate and the adsorption duration compared to the calcium alginate hydrogels without algae loading. Notably, at higher initial concentrations of metal ions, the algae-enriched composite adsorbents exhibited a more pronounced enhancement in adsorption efficiency. Furthermore, the composite adsorbents maintained stability post-adsorption, thereby minimizing the ecological risks associated with the use of algae biomass in aquatic systems. In conclusion, we have developed a promising, environmentally friendly, and sustainable alginate-based hydrogel adsorbent that offers a highly efficient solution for the removal of heavy metal ions from contaminated water.

## 4. Materials and Methods

### 4.1. Materials

Sodium alginate and calcium chloride were purchased from ADAMAS-BETA Shanghai Luchi Trading Co., Ltd. (Beijing, China). Cobalt (II) nitrate hexahydrate, chromium (III) nitrate nonahydrate, and copper (II) sulfate were purchased from Sinopharm Chemical Reagent Co., Ltd. (Shanghai, China). Lyophilized Chlorella vulgaris powder was obtained from Shanghai Guangyu Biological Technology Co., Ltd. (Shanghai, China). All chemicals were used as received without further purification.

### 4.2. Hydrogel Preparation

The calcium alginate hydrogel was prepared by calcium-induced crosslinking according to the reported method with slightly modification [27]. Firstly, sodium alginate was dissolved in deionized water to achieve a 2.0% (*w*/*v*) solution. To introduce algae into the hydrogel matrix, varying amounts (1 mg, 2 mg, and 5 mg, respectively) of Chlorella vulgaris powder were added to 1 mL of sodium alginate solution. Then, 1 mL of the prepared sodium alginate–algae mixture was then added dropwise to 1 mL of 0.1 M calcium chloride (CaCl_2_) solution. The resulting mixtures were allowed to stand at 25 °C for 10 min, during which the gelation of the sodium alginate was initiated by the calcium ions. After the gelation process, the hydrogel was carefully removed and washed three times with deionized water to remove any residual calcium chloride. The washed hydrogels were then stored at room temperature or lyophilized for subsequent analysis. In addition, the calcium alginate hydrogel was prepared as a blank control without algae addition, using the same preparation method as above.

### 4.3. Scanning Electron Microscopy (SEM)

The morphology of the prepared hydrogels was examined using a Zeiss Sigma 300 VP Scanning Electron Microscope (SEM) (Oberkochen, Germany). The samples were gold-coated to improve conductivity and observed under secondary electron mode at an accelerating voltage of 2 kV.

### 4.4. Fourier Transform Infrared Spectroscopy (FTIR)

The chemical structure and functional groups of the hydrogels were characterized using Fourier Transform Infrared Spectroscopy (FTIR) with a Thermo-Nicolet iS5 spectrometer (Waltham, MA, USA). The hydrogel samples were first frozen and lyophilized in polyethylene tubes. After lyophilization, the samples were finely ground and placed on the FTIR sample holder. The spectra were recorded in the range of 4000 to 400 cm^−1^. To ensure accuracy, the absorption spectra were corrected by subtracting the baseline data from a potassium bromide (KBr) blank sample.

### 4.5. Confocal Laser Scanning Microscopy (CLSM)

To evaluate the distribution of Chlorella vulgaris in the calcium alginate hydrogels, Confocal Laser Scanning Microscopy (CLSM) was performed using a Nikon Ti2 confocal microscope (Nikon, Tokyo, Japan). Auto-fluorescent Chlorella facilitated the visualization in the hydrogels. The samples were prepared by placing the hydrogel slices onto the microscope slides, and the images were captured at different focal planes to assess the uniformity and dispersion of the algae throughout the hydrogel matrix. CLSM images were processed using Nikon NIS-Elements AR software 5.21.00 to analyze the distribution patterns.

### 4.6. Inductively Coupled Plasma-Optical Emission Spectroscopy (ICP-OES)

The concentrations of heavy metal ions in solution after the adsorption experiments were measured using Inductively Coupled Plasma–Optical Emission Spectroscopy (ICP-OES, Leeman Prodigy, Hudson, NH, USA). The metal ions were quantified by comparing the emission spectra to calibration standards for copper (Cu^2+^), chromium (Cr^3+^), and cobalt (Co^2+^).

### 4.7. Adsorption Experiments

To evaluate the heavy metal ion adsorption capacity of the algae-containing calcium alginate hydrogels, adsorption experiments were conducted using Cu^2+^, Cr^3+^, and Co^2+^ as model heavy metal ions. All metal ion solutions were prepared by dissolving metal ions in deionized water. About 20 mg of hydrogels (both with and without Chlorella) were immersed in 20 mL solutions of these three metal ions at an initial concentration of 100 ppm, respectively. The adsorption experiment was performed at 25 °C with stirring at 100 rpm. After 12 h of agitation at room temperature, the remaining concentrations of metal ions in the solution were measured using ICP-OES. The difference in metal ion concentrations before and after adsorption allowed for the calculation of the adsorption capacity of the hydrogels. The adsorption capacities were calculated as follows: adsorption capacity = (mass of metal in solution before adsorption − mass of metal in solution after adsorption)/mass of hydrogel.

For adsorption kinetics, about 20 mg of hydrogels (both with and without Chlorella) were immersed in 20 mL solutions of Cu^2+^, Cr^3+^, and Co^2+^ at an initial concentration of 100 ppm, respectively. Then, the metal ion concentrations in the solutions were measured at different time intervals (at 0, 0.5, 1, 2, 3, 4, 5, 6, 7, 8, 9, 10, 11, and 12 h). The adsorption experiment was performed at 25 °C with stirring at 100 rpm.

To study the effect of initial metal ion concentration on the adsorption capacity, additional experiments were performed using metal ion solutions (Cu^2+^, Cr^3+^, and Co^2+^) with different initial concentrations. About 20 mg of the hydrogels (both with and without Chlorella) were immersed in 20 mL solutions at initial metal ion concentrations of 100 ppm, 200 ppm, 500 ppm, and 1000 ppm, respectively. After 12 h of incubation, the final concentrations of metal ions in each solution were determined, and the adsorption capacity was calculated based on the decrease in concentration. The adsorption experiment was performed at 25 °C with stirring at 100 rpm.

## Data Availability

The data presented in this study are available on request from the corresponding authors.
